# Clinical Characteristics of Lamellar Macular Hole Subtypes: Degenerative and Tractional

**DOI:** 10.1155/2021/5610199

**Published:** 2021-11-20

**Authors:** Joseph Kim, Joon Ki Min, Se Young Kim, Myung Hun Yoon, Hee Seung Chin

**Affiliations:** ^1^Department of Ophthalmology, Inha University School of Medicine, Incheon, Republic of Korea; ^2^Retina Division, Nune Eye Hospital, Seoul, Republic of Korea; ^3^Blue Eye Center, Incheon, Republic of Korea

## Abstract

**Purpose:**

To analyze clinical parameters of two subtypes of lamellar macular hole (LMH): degenerative and tractional.

**Methods:**

This retrospective chart review study included patients monitored for more than 6 months after the initial diagnosis of LMH from January 2011 to January 2018. LMH was classified in two subtypes: degenerative and tractional. The following parameters between both subtypes were assessed: central subfield thickness (CST), maximum inner diameter (MID), maximum outer diameter (MOD), MID/MOD ratio, inner and outer segment (IS/OS) junction disruption, residual retinal thickness (RRT), subfoveal choroidal thickness (SFCT), best-corrected visual acuity (BCVA), anatomical progression rate, and percentage of patients undergoing surgery.

**Results:**

This study included 51 eyes with a mean follow-up period of 18.94 months: 33 eyes with tractional LMH and 18 eyes with degenerative LMH. MID was not significantly different between both subtypes but MOD was significantly greater in tractional LMH than degenerative types (tractional, 1131.6 *μ*m; degenerative, 708.9 *μ*m; *p* < 0.001). The MID were significantly increased in degenerative eyes, while the tractional eyes featured a significant increase in MOD. BCVA was not significantly different between both subtypes at baseline and the last follow-up. Epiretinal membrane presence was significantly different between the two subtypes (tractional, 96.9%; degenerative, 22.2%; *p* < 0.001). Ellipsoid defect and rate of receiving surgery were not significantly different between both subtypes. The anatomical progression rate in tractional eyes (81.8%) was significantly higher than that of degenerative LMH (27.7%) (*p* = 0.010). The SFCT was correlated to anatomical progression in the tractional LMH (correlation coefficient = 0.351, *p* = 0.049) but not in the degenerative LMH. During the follow-up period, 4 eyes (22.2%) of the degenerative LMH and 11 eyes (33.3%) of the tractional LMH underwent surgery.

**Conclusions:**

We found that greater SFCT at baseline was correlated to anatomical progression of tractional LMH. Therefore, it is expected that SFCT could be used as a biomarker to predict anatomical progression in tractional LMH.

## 1. Introduction

Lamellar macular hole (LMH) is a partial-thickness macular defect with an irregular foveal contour and intraretinal splitting [1]. In 1976, Gass and Norton first reported LMH with a surface macular lesion of a multilobulated cystic macular edema on conventional biomicroscopy [2]. LMH is currently diagnosed using optical coherence tomography (OCT). Improvement in OCT resolution revealed LMH to be highly heterogeneous in morphology [1]. Govetto et al. proposed two different types of LMH based on spectral domain OCT findings: (1) tractional LMH, which is thought to be caused by horizontal forces exerted by a classic epiretinal membrane (ERM), and (2) degenerative LMH, which is clearly distinguished by the lack of a traction element and has nontractional lamellar hole-associated epiretinal proliferation (LHEP) [3, 4]. The surgical benefits for degenerative LMH with LHEP have been reported to be more limited than those for tractional LMH [5–8]. Recently, it has been reported that thin choroidal thickness is associated with LMH [9]. LMH is still an area that needs to be further understood. The purpose of this study is to analyze the clinical characteristics of LMH between two subtypes (tractional and degenerative) to further understand the progression and prognosis of this disease.

## 2. Methods

This retrospective chart review study adhered to the tenets of the Declaration of Helsinki and received approval from the Inha University Hospital Institutional Review Board/Ethics Committee. We reviewed the medical records of patients who were diagnosed with LMH at Inha University Hospital from January 2011 to January 2018 with more than 6 months of follow-up data. The exclusion criteria were myopia of more than 6 diopters, history of vitreoretinal surgery, and presence of any other retinal disease.

**Figure 1:**
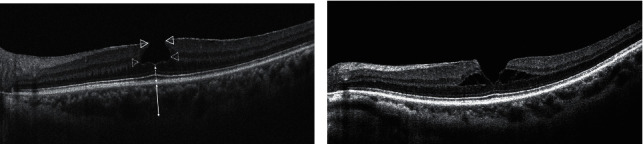
Measurement using the caliper function. (a) Degenerative lamellar macular hole: maximum inner diameter, outlined arrowhead; maximum outer diameter, dotted arrowhead; residual retinal thickness, dotted line with double arrowheads; subfoveal choroidal thickness, solid line with double arrowheads. (b) Tractional lamellar macular hole.

Spectral domain OCT enhanced depth images were obtained using the Cirrus HD-OCT 4000 (Carl Zeiss Meditec AG, Oberkochen, Germany) with macular cube (512 × 128) and HD 5-line raster (angle: 0°, spacing: 0.5 mm, length: 9 mm) protocol and analyzed using the Cirrus review software version 6.0. All patients were classified into two subtypes (tractional and degenerative) according to the classification by Govetto et al. [3]. Tractional LMH was characterized by intraretinal schisis, hyperreflective bridges, and moustache appearance in the presence of tractional ERM. Degenerative LMH was characterized by a top-hat appearance with round-edged intraretinal cavitation, central retinal bump, and presence of epiretinal proliferation. The following OCT characteristics were analyzed as the clinical parameters: central subfield thickness (CST), maximum inner diameter (MID), maximum outer diameter (MOD), MID/MOD ratio, and inner and outer segment (IS/OS) junction disruption. The CST was automatically measured using a thickness map within an inner 1 mm diameter circle. The other OCT parameters were manually measured on the line scan image crossing the fovea using the caliper function which was built in Cirrus review software version 6.0. The MID was measured as the longest length of the ILM level, and the MOD was measured as the longest length in the intraretinal cavity. The residual retinal thickness (RRT) was measured from the vitreoretinal surface to the retinal pigment epithelium (RPE) at the base of the LMH. The subfoveal choroidal thickness (SFCT) was measured from the outer part of the hyperreflection line corresponding to the RPE to the inner surface of the sclera (Figure 1). The clinical parameters also included the best-corrected visual acuity (BCVA), anatomical progression rate, and percentage of patients undergoing surgery. Anatomical progression was defined as an increase of more than 50 *µ*m of the inner or outer diameters from baseline or the appearance of a full-thickness macular hole [3].

Statistical analysis was performed using the SPSS software version 18 (SPSS Inc., Chicago, IL, USA). Comparisons of the parameters between the LMH subtypes were performed using the Mann–Whitney *U* test. Changes in the parameters from baseline to the end of follow-up were assessed using the Wilcoxon signed-rank test. Pearson correlation coefficient was used to correlate clinical factors with anatomical progression. The significance level was set at *p* < 0.05.

## 3. Results

Of the 80 eyes with LMH diagnosed during the study period, 51 eyes of 49 patients met the inclusion criteria and were enrolled in the study. 39 eyes were excluded due to follow-up of less than 6 months. The mean age of the enrolled patients was 66.37 ± 10.79 years. Eighteen and thirty-three eyes were classified into the degenerative and tractional LMH, respectively. The mean age of the patients in the tractional and degenerative LMH was similar: 65.32 ± 11.72 and 68.72 ± 8.18 years, respectively (*p* = 0.491). The sex ratio was significantly different between the two subtypes. The tractional LMH featured more women than did the degenerative (*p* < 0.001). The MID/MOD ratio (below 0.5) and presence of ERM were higher in the tractional type than in the degenerative type (MID/MOD ratio (below 0.5): tractional LMH (63.6%) and degenerative (11.1%), *p* < 0.001; presence of ERM: tractional LMH (96.9%) and degenerative (22.2%), *p* < 0.001). LHEP was observed in thirteen eyes only in the degenerative LMH (72.2%). IS/OS junction disruption was found in two eyes in each subtype, and no additional occurrence was observed during the follow-up period (Table 1).

At baseline, there were no differences found in the CST, MID, RRT, SFCT, and BCVA between the two subtypes. However, the MOD was greater in the tractional LMH (*p* < 0.001, Table 2). The CST did not increase significantly across the follow-up period in both types (tractional: 319.4 ± 53.3 *μ*m at baseline versus 324.6 ± 51.5 *μ*m at the last follow-up, *p* = 0.508; degenerative: 300.0 ± 64.8 *μ*m at baseline versus 300.8 ± 70.9 *μ*m at the last follow-up, *p* = 0.931; Table 2). The increases in the MID and MOD differed according to the subtypes. In the tractional LMH, while the mean MID did not change significantly during the last follow-up (*p* = 0.338), the MOD increased significantly across the follow-up period (*p* = 0.003). In the degenerative type, the mean MOD did not change significantly across the follow-up period (*p* = 0.231), but the mean MID significantly increased by the last follow-up (*p* = 0.045). The BCVA did not change across the follow-up period in both subtypes (tractional: *p* = 0.532; degenerative: *p* = 0.759). The anatomical progression rate was much higher in the tractional group than in the degenerative group (tractional LMH: 81.8%; degenerative LMH: 27.7%; *p* = 0.010, Table 2). During the follow-up period, 4 eyes in the degenerative LMH and 11 eyes in the tractional LMH underwent surgery. Surgery was performed at the discretion of the clinician; however, further analysis was not possible because detailed evidence for the decision to operate was not recorded.

A subgroup analysis was performed to assess the correlation between the baseline parameters and anatomical progression. The SFCT was found to be associated with anatomical progression in the tractional LMH (correlation coefficient = 0.351, *p* = 0.049) but not with that in the degenerative LMH (correlation coefficient = −0.023, *p* = 0.929). The CST, MID, MOD, and RRT were not found to be correlated with anatomical progression in either the degenerative group (*p* = 0.744, 0.075, 0.104, 0.894, and 0.943, respectively) or the tractional group (*p* = 0.131, 0.319, 0.964, 0.501, and 0.832, respectively; Table 3).

## 4. Discussion

In this study, clinical parameters of two lamellar macular hole subtypes were analyzed. Govetto et al. [3] suggested that the presence of traction and vitreomacular traction (VMT) may play an important role in tractional LMH development and that the schisis-like separation is a response to the tractional stress. Meanwhile, the degenerative LMH may be caused by a different pathway which is a slow, chronic, degenerative process causing loss of retinal tissue rather than a definite schisis-like separation.

We found an interesting result in SFCT of lamellar macular hole subtypes. A greater SFCT at baseline was correlated with anatomical progression in the tractional LMH. We assumed that the anatomical progression was related to tractional force and VMT and that the greater SFCT might be caused by the greater tractional force and VMT. In a previous study of Fang and Chen [10], choroidal thickness in the ERM eye was greater than in the fellow healthy eye and choroidal thickness in the ERM eyes decreased after ERM removal surgery. They insisted that ERM contraction force caused an increase in choroidal thickness. In another ERM study with and without VMT, the SFCT was greater in an ERM with VMT. The authors suggested that anteroposterior (AP) force induced by VMT was associated with an increase in the choroidal thickness [11]. AP traction on the retina can affect the RPE and choroid because a force is always met by an equal force in the opposite direction. Meanwhile, RPE stretching can increase the level of the vascular endothelial growth factor (VEGF). Elevated VEGF levels lead to hyperpermeability of the choroidal vessels and subsequent choroidal thickening [11–13]. Therefore, we could conclude that the greater SFCT in tractional LMH reflects greater tractional force and VMT. The tractional type often accompanies an ERM, and our study also found an ERM (96.9%) in the tractional LMH. An ERM is generally associated with tangential traction that distorts the inner retinal configuration [14, 15] and also causes AP and centripetal traction [16]. Therefore, we can reasonably infer that the greater SFCT in tractional LMH is attributed to the large tangential traction and AP stress, and in that situation, more anatomical progression may occur. Conversely, in degenerative LMH, which is believed to be another entity, the SFCT was not associated with anatomical progression.

Recently, Kal et al. [9] reported a lower choroidal thickness in patients with LMH than in healthy subjects. This study presented that thinning choroid might be involved in the development of LMH. The authors suggested that the choroid could play a role in the pathogenesis of LMH and that significant choroidal thinning could be responsible for the ischemic degenerative mechanism of the outer layers of the retina in LMH. Choi et al. [17] reported thin choroid thickness in the ERM eye and choroidal thickening in the spontaneously resolved ERM eye. These findings appear to be in conflict with our result. So far, studies on the LMH have not shown consistent results on the association between LMH and choroidal thickness. Hence, further studies are required to reveal more clear correlations between LMH and choroidal thickness.

Meanwhile, our study found that the anatomical progression patterns were different between degenerative and tractional LMH. In the degenerative type, the MID increased over time, whereas in the tractional type, the MOD predominantly increased. It can be understood that the MOD may be more affected in tractional LMH because the morphological characteristic of the tractional type is the separation and progression between the outer plexiform and the outer nuclear layers. In the degenerative type, it is estimated that the retinal tissue is lost through a chronic degenerative process. A previous study reported that both MID and MOD significantly increased during the follow-up period in degenerative LMH [3]. Therefore, it is premature to understand and conclude which of the MID and MOD will indicate the dominant progression in the degenerative type.

The limitations of our study include its retrospective nature and small sample size which make it difficult to perform adequate power calculations. Manual measurements using the caliper function of OCT may inherently yield measurement bias. Nevertheless, we attempted to minimize the error through cross-checking. Another limitation is that we did not consider diurnal variations when SFCT was measured. These measurements should be ideally performed in the same period of the day.

## 5. Conclusion

This study found a correlation between LMH progression and the SFCT in the tractional LMH. In the degenerative LMH, there was no significant association between disease progression and the choroidal thickness. Therefore, we anticipate that SFCT could be used as a biomarker to predict anatomical progression of tractional LMH.

## Figures and Tables

**Table 1:** Baseline characteristics.

Factors	Degenerative LMH (*n* = 18)	Tractional LMH (*n* = 33)	*P* value^*∗*^
Sex	Male: 11 (61.1%) Female: 7 (38.9%)	Male: 4 (12.1%) Female: 29 (87.9%)	<0.001
Age, year	68.72 ± 8.18	65.32 ± 11.72	0.491
Mean follow-up months	23.27 ± 22.11	16.57 ± 12.52	0.102
ERM	4 (22.2%)	32 (96.9%)	<0.001
LHEP	13 (72.2%)	0%	0.017
IS/OS junction disruption	2 (11.1%)	2 (6%)	0.526
MID/MOD ratio of <1 : 2	11.1%	63.6%	0.001

^*∗*^Mann–Whitney *U* test, ERM, epiretinal membrane; LHEP, lamellar hole-associated epiretinal proliferation; IS/OS, inner and outer segment; MID, maximum inner diameter; MOD, maximum outer diameter.

**Table 2:** Anatomical and clinical characteristics of degenerative and tractional LMH.

Factors		Degenerative LMH	Tractional LMH	*P* value^†^
CST	Baseline	300.0 ± 64.8 *μ*m	319.4 ± 53.3 *μ*m	0.141
	End of follow-up	300.8 ± 70.9 *μ*m	324.6 ± 51.5 *μ*m	0.092
		*P* = 0.931^*∗*^	*P* = 0.508^*∗*^	

MID	Baseline	654.2 ± 166.1 *μ*m	572.7 ± 197.6 *μ*m	0.241
	End of follow-up	730.6 ± 212.0 *μ*m	602.88 ± 201.7 *μ*m	0.070
		*P* = 0.045^*∗*^^,‡^	*P* = 0.338^*∗*^	

MOD	Baseline	708.9 ± 375.2 *μ*m	1131.62 ± 433.5 *μ*m	<0.001^‡^
	End of follow-up	697.8 ± 292.6 *μ*m	1358.2 ± 604.5 *μ*m	<0.001^‡^
		*P* = 0.231^*∗*^	*P* = 0.003^*∗*^^,‡^	

BCVA, logMAR (Snellen equivalent)	Baseline	0.147 ± 0.136 (20/28)	0.224 ± 0.210 (20/33)	0.193
	End of follow-up	0.156 ± 0.189 (20/29)	0.239 ± 0.219 (20/35)	0.091
		*P* = 0.759^*∗*^	*P* = 0.532^*∗*^	

RRT	Baseline	133.7 ± 45.1 *μ*m	152.7 ± 37.5 *μ*m	0.246

SFCT	Baseline	192.4 ± 81.9 *μ*m	219.8 ± 64.9 *μ*m	0.106

Anatomical progression rate		5 (27.7%)	27 (81.8%)	0.934

^*∗*^Wilcoxon signed-rank test, ^†^Mann–Whitney *U* test, ^‡^Statistically significant. CST, central subfield thickness; LMHs, lamellar macular holes; MID, maximum inner diameter; MOD, maximum outer diameter; BCVA, best-corrected visual acuity; RRT, residual retinal thickness; SFCT, subfoveal choroidal thickness.

**Table 3:** Subgroup analysis to find clinical factors correlated to anatomical progression.

	Degenerative LMH coefficient^*∗*^ (*p* value)	Tractional LMH coefficient^*∗*^ (*p* value)
BCVA	−0.083 (0.744)	0.268 (0.131)
CST	0.515 (0.075)	−0.179 (0.319)
MID	−0.396 (0.104)	0.008 (0.964)
MOD	0.034 (0.894)	0.122 (0.501)
RRT	0.020 (0.943)	0.040 (0.832)
SFCT	−0.023 (0.929)	0.351 (0.049)^†^

^*∗*^Pearson correlation coefficient.

## Data Availability

The data used to support the findings of this study are available from the corresponding author upon request.
